# Biopsy-Based Transcriptomics Support Rejection Monitoring Through Repeated Kidney Allograft Biopsies

**DOI:** 10.1016/j.ekir.2025.04.043

**Published:** 2025-04-27

**Authors:** Lukas Weidmann, Dusan Harmacek, Ariana Gaspert, Birgit Maria Helmchen, Britta George, Kerstin Hübel, Seraina von Moos, Elena Rho, Thomas Schachtner

**Affiliations:** 1 Division of Nephrology, University Hospital of Zurich, Switzerland; 2 Division of Pathology and Molecular Pathology, University Hospital of Zurich, Switzerland; 3 Division of Nephrology, Cantonal Hospital of Lucerne, Switzerland

**Keywords:** antirejection treatment, biopsy-based transcriptomics, kidney allograft rejection, molecular diagnostics

## Abstract

**Introduction:**

Biopsy-based transcriptomics may detect subthreshold signals suspicious for rejection in histologically “rejection-free” biopsies, reflect antirejection treatment responses, and indicate gradual phenotyping of rejection in kidney allograft biopsies.

**Methods:**

We investigated 80 “biopsy series” (baseline and corresponding follow-up biopsies) from 2018 to 2025, assessed by histopathology (Banff classification) and the Molecular Microscope Diagnostic System (MMDx).

**Results:**

Baseline biopsies showed histological rejection, including partial antibody-mediated rejection (AMR) and borderline T-cell–mediated rejection (TCMR) in 55 of 80 cases (69%), with 55% molecular rejection confirmation. After a median of 9 months (interquartile range: 4–18), follow-up biopsies detected histological rejection in 9 of 25 (36%) previously “rejection-free” cases. Corresponding baseline biopsies had higher rejection (Rejection_prob_; median 0.23 vs. 0.02, *P* = 0.008) and AMR (AMR_prob_; 0.08 vs. 0.03, *P* = 0.002) classifier scores than those without follow-up rejection (*n* = 16). Histological interstitial inflammation (i) + tubulitis (t) and glomerulitis (g) + peritubular capillaritis (ptc) scores were similar (*P* = 0.411, *P* = 0.602). In molecular TCMR treated conventionally, 4 of 6 (67%) had TCMR_prob_ < 0.1 at follow-up, with significant reductions in i+t scores (4.5–1.5, *P* = 0.031). Two of 6 (33%) progressed to mixed phenotypes. Among treated molecular AMR cases, AMR_prob_ decreased (0.76–0.51, *P* = 0.047), but only 1 of 7 (14%) reached AMR_prob_ < 0.2, whereas g + ptc scores remained unchanged (*P* > 0.99). In treated mixed molecular AMR/TCMR, molecular and histological scores improved. Four of 9 (44%) showed rejection resolution, 3 of 9 (33%) shifted to molecular AMR, 1 of 9 (11%) to TCMR, and 1 of 9 (11%) remained mixed molecular phenotypes.

**Conclusions:**

Biopsy-based transcriptomics differentiated suspicious molecular signals among histologically “rejection-free” biopsies progressing to rejection and provided a monitoring tool after antirejection treatment interventions.

## Graphical abstract

**Figure:**
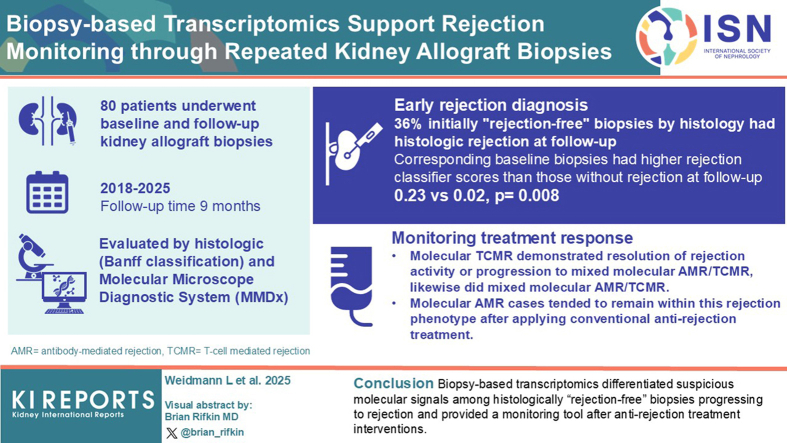

Histological diagnosis of kidney allograft rejection using the Banff classification can be subjective because of semiquantitative lesion-scoring.^1^^,^^2^ In response, biopsy-based transcriptomics such as the MMDx have been developed to reduce interobserver variability and more objectively classify rejection phenotypes.^3^ Although MMDx received validation across various studies, including our own cohort, its added value as a follow-up tool in repeat biopsies, especially following antirejection treatment, remains underexplored.^4^, ^5^, ^6^, ^7^, ^8^ The potential of molecular diagnostics to reflect treatment responses has been highlighted before, mostly within scenarios of chronic-active AMR or specific situations such as the conversion to belatacept.^9^, ^10^, ^11^, ^12^ Moreover, it has been shown that MMDx can detect rejection signs earlier than histology, or discussed that it could classify rejection gradually along the AMR and TCMR continuum.^13^^,^^14^ The Banff update from 2022 introduces the following 2 new entities into the AMR continuum: (i) “Probable AMR” consists of cases with donor-specific antibodies (DSAs) and active or chronic histological lesions for AMR, not reaching significance for the full histological diagnosis of AMR (i.e., microvascular inflammation [MVI] below threshold); and (ii) “MVI, DSA-negative, and C4d-negative” includes cases with relevant MVI (at or above threshold), but without DSA or C4d-positivity.^15^ The Banff consortium encourages clinicians to incorporate MMDx in these ambiguous biopsy cases, because it serves as a potential substitute for MVI (especially relevant in probable AMR). Furthermore, follow-up in such scenarios is crucial, because recent evidence highlighted that both “probable AMR” and “MVI, DSA-negative, and C4d-negative” can progress to AMR and are associated with adverse outcomes.^16^^,^^17^ Of note, “MVI, DSA-negative, and C4d-negative” has been proposed as a form of AMR without detectable circulating DSA at the time of biopsy, indicating that DSA might have fully bound to the allograft.^17^ However, its pathophysiology remains not fully understood, because previous work demonstrated links between DSA-negative MVI and nonhumoral missing self-mechanisms involving natural killer cells.^18^ More recently published investigations even described associations between MVI and T-cell–mediated processes.^19^ Under these circumstances, transcriptomics could serve as a crucial guide, helping to determine whether the development is leaning toward rejection or not. Gradual rejection phenotyping might become more relevant in the future. In this context, a recent study found that subthreshold molecular TCMR activity is present in many AMR biopsies and subthreshold molecular AMR activity in many TCMR biopsies.^14^ In addition, this study demonstrated that some biopsies without histologically proven rejection but with subthreshold MMDx lesions (especially molecular classifiers) tend to progress to full rejection in follow-up biopsies.^14^ In our cohort, we aimed to evaluate the potential of MMDx to detect subthreshold molecular rejection signs in histologically “rejection-free” biopsies, reflect antirejection treatment responses, and classify rejection phenotypes more gradually along the AMR and TCMR continuum in 80 baseline and corresponding follow-up biopsies.

## Methods

### Cohort Definitions and Ethics

Eighty patients with 160 unselected, prospectively conducted allograft biopsies were assessed between October 2018 and January 2025 and extracted from our MMDx biopsy cohort, consisting of 478 biopsies performed between July 2018 and January 2025. Each patient had 2 biopsies (baseline biopsy and corresponding follow-up biopsy) assessed by histology and MMDx. Each consecutive pair of biopsies was considered a “biopsy series.” The detailed deduction is demonstrated in Supplementary Figure S1. All patients provided general consent for using their clinical data, including biopsy results and MMDx analysis. The investigation was approved by the cantonal ethics commission review board of Zurich, Switzerland (BASEC 2020-02817) and has complied with the Declaration of Helsinki.

### Allograft Biopsy Policy and Processing

At our center, only ABO-incompatible transplantations and a subset of ABO-compatible transplantations received protocol biopsies at month 3 and month 12 after transplantation. Biopsy indications were not counted as protocol biopsies if there was a clinical indication (decline of kidney function, de novo DSA, and/or proteinuria). We started collecting biopsy samples prospectively in 2018, preserving them in RNAlater solution. From 2021 onward, both newly obtained and previously stored biopsies (dating back to 2018) were sent to the MMDx provider for analysis. Allograft biopsies were evaluated at the bedside for adequacy (sufficient cortex) during the procedure. Local nephropathologists assigned histopathological diagnoses according to the Banff classification, last updated in 2022.^15^^,^^20^ All biopsies that had been performed at earlier time points were subsequently reevaluated and graded according to the Banff 2022 criteria. Small tissue samples (3–4 mm) were obtained from the biopsy cores for molecular evaluation. MMDx analysis was performed per protocol in Kashi Clinical Laboratories (Portland, OR).

### Histological Categorization and DSAs

Histological rejection included AMR (active and chronic-active), “MVI, DSA-negative, C4d-negative”, TCMR (grade IA–III), and mixed AMR/TCMR, with mixed cases requiring full histological criteria for both AMR and TCMR. Probable AMR and borderline TCMR changes were classified as “subthreshold histological rejection lesions”, because Banff lesion scores remained below cutoffs and DSA-positivity does not count as a histological criterion. MVI was recorded as below threshold (g + ptc = 1) or at/above threshold (g + ptc ≥ 2), with isolated ptc lesions counted only if unrelated to borderline TCMR, TCMR, or infection. Isolated mild intimal arteritis lesions (v1 lesions) were not counted as rejection in the absence of at least borderline inflammation (i > 0 and t > 0) or AMR. C4d was evaluated via immune fluorescence and interpreted as accommodation in cases with ABO-incompatible transplantation. DSAs were assessed using OneLambda single antigen beads, with a detection cutoff of MFI > 500. Both preformed DSAs and *de novo* DSAs were analyzed.

### MMDx Categorization and Analysis

Each biopsy was molecularly classified using AMR (AMR_prob_) and TCMR (TCMR_prob_) classifier scores, resulting in 4 distinct subgroups as follows: (i) mixed molecular AMR/TCMR defined as AMR_prob_ > 0.2 and TCMR_prob_ > 0.1; (ii) molecular AMR defined as AMR_prob_ > 0.2 and TCMR_prob_ < 0.1; (iii) molecular TCMR defined as AMR_prob_ < 0.2 and TCMR_prob_ > 0.1; and (iv) no molecular rejection defined as AMR_prob_ < 0.2 and TCMR_prob_ < 0.1. The upper limit of normal for the rejection classifier (Rejection_prob_), AMR_prob_, and TCMR_prob_ was 0.3, 0.2, and 0.1, respectively, as defined by the validation center. Subthreshold cutoffs for this study were set at 0.15, 0.1, and 0.05, respectively. In addition to molecular rejection classifiers, we examined injury scores, including global disturbance, acute kidney injury (AKI), and atrophy/fibrosis. Furthermore, we incorporated archetypal analysis in some of the investigations, which is represented by the following 6 archetype scores: (i) R1 (non-rejecting), (ii) R2 (TCMR2), (iii) R3 (TCMR1, formerly mixed), (iv) R4 (early-stage AMR), (v) R5 (fully-developed AMR), and (vi) R6 (late-stage AMR).

### Assessment of Antirejection Treatments

Conventional antirejection treatments consisted of pulsed steroids, defined as 3 to 5 doses of 250 mg or 500 mg of methylprednisolone, antithymocyte globulin, plasmapheresis with i.v. Ig; and rituximab. Changes in histological and molecular markers were considered. Only cases with molecular rejection were investigated in depth. Antiinterleukin 6 therapies (tocilizumab, clazakizumab) were analyzed in a subgroup of molecular AMR (*n* = 10). Treatment decisions were not prespecified, but made on a case-by-case basis, guided by the clinical context.

### Statistical Analysis

Statistical analysis was performed with GraphPad Prism Version 10.4.1. Nonparametric tests, including the Mann-Whitney U or the Wilcoxon signed rank-test, were used to compare continuous variables, as appropriate. Categorical variables were compared using chi-square or Fisher exact test. Probability values and confidence intervals were 2-sided. Continuous variables were reported as median and interquartile range (Q1–Q3) or mean ± SD, regardless of distribution. Categorical variables were presented as numbers (*n*) and percentages (%). A *P*-value < 0.05 was considered statistically significant.

## Results

### Basic Characteristics

Of the cohort, 34% were female, with a median (interquartile range) age of 44 (33–53) years at transplantation. The median latency between the baseline and follow-up biopsies was 9 (4–18) months. Of the baseline biopsies, 53% were conducted because of a decline in estimated glomerular filtration rate, and 9% were protocol biopsies. The basic characteristics are demonstrated in Table 1 with additional information presented in Supplementary Table S1.

**Table 1:** Basic and biopsy characteristics

Demographics and HLA		
	All patients (*N* = 80)	
Female sex, *n* (%)	27 (34%)	
Age at TPL (years), median (IQR)	44 (33–53)	
Living donation, *n* (%)	37 (46%)	
Repeat TPL, *n* (%)	6 (8%)	
Time between TPL and baseline bx (mos), median (IQR)	15 (3–68)	
Time between baseline and follow-up bx (mos), median (IQR)	9 (4–18)	
HLA mismatches ^3^ 5, *n* (%)	33 (41%)	

Biopsy information		

	Baseline (*n* = 80)	Follow-up (*n* = 80)

DSA-presence at bx		
pDSA, *n* (%)	13 (16%)	12 (15%)
dnDSA, *n* (%)	29 (36%)	37 (46%)
Bx indication^a^		
DSA, *n* (%)	37 (46%)	44 (55%)
eGFR decline, n (%)	42 (53%)	32 (40%)
Proteinuria rise, n (%)	20 (25%)	17 (21%)
Protocol bx, n (%)	7 (9%)	7 (9%)
Histological categorization		
Mixed AMR/TCMR, n (%)	3 (4%)	1 (1%)
AMR, n (%)	16 (20%)	26 (33%)
AMR (active), n (%)	3 (4%)	8 (10%)
AMR (chronic-active), n (%)	13 (16%)	18 (23%)
MVI, DSA-negative, and C4d-negative, n (%)	11 (14%)	9 (11%)
TCMR, n (%)	11 (14%)	7 (6%)
Grade IA/IB, n (%)	0 (0%)	1 (1%)
Grade IIA/IIB/III, n (%)	11 (14%)	6 (8%)
Subthreshold histological rejection lesions, n (%)	14 (18%)	10 (13%)
Borderline TCMR changes, n (%)	3 (4%)	0 (0%)
Probable AMR, n (%)	11 (14%)	10 (13%)
No histological rejection, n (%)	25 (31%)	27 (34%)
Relevant tubulitis (t≥2), n (%)	4 (5%)	4 (5%)
MVI 1 (g+ptc=1) without DSA, n (%)	4 (5%)	9 (11%)
Recurrent or *de novo* disease, n (%)	2 (3%)^b^	5 (6%)^c^
Molecular categorization		
Mixed molecular AMR/TCMR, n (%)	11 (14%)	9 (11%)
Molecular AMR, n (%)	18 (23%)	25 (31%)
Molecular TCMR, n (%)	8 (10%)	4 (5%)
No molecular rejection, n (%)	43 (54%)	42 (53%)

AMR, antibody-mediated rejection; bx, biopsy; dnDSA, *de novo* DSA; DSA, donor-specific antibody; eGFR, estimated glomerular filtration rate; FSGS, focal segmental glomerulosclerosis; HLA, human leukocyte antigen; IQR, Interquartile range; MVI, microvascular inflammation; pDSA, preformed DSA; TCMR, T cell–mediated rejection; TPL, transplantation.

In cases with glomerulonephritis, glomerulitis (g) was not counted as part of MVI.

Continuous variables are reported as median and IQR; and categorical variables as numbers (*n*) and percentages (%).

a One biopsy could have more than one indication.

b One case of IgA nephropathy and one case of FSGS.

c One case of IgA nephropathy, one case of FSGS, one case of lupus nephritis, one case of C1q nephropathy and one case of acute tubulointerstitial nephritis.

### Categorization and Molecular Confirmation of Rejection Subgroups

Three of 80 baseline biopsies (4%) were histologically diagnosed as mixed AMR/TCMR; 16 of 80 (20%) as AMR; 11 of 80 (14%) as MVI, DSA-negative, and C4d-negative; 11 of 80 (14%) as TCMR; 14 of 80 (18%) as subthreshold histological rejection lesions (11 cases with probable AMR, 3 cases with borderline TCMR changes); and 25 of 80 (31%) as no rejection. Detailed characteristics of the histological subgroups are presented in Supplementary Table S2. Molecularly, 11 of 80 (14%) were diagnosed as mixed molecular AMR/TCMR, 18 of 80 (23%) as molecular AMR, 8 of 80 (10%) as molecular TCMR, and 4 of /80 (54%) as no molecular rejection. At follow-up, the proportion of mixed AMR/TCMR, TCMR, and subthreshold histological lesions based on histology declined (1%, 6%, and 13% at follow-up, respectively), whereas the proportion of AMR increased (33% at follow-up). Molecularly, the percentage of mixed molecular AMR/TCMR and molecular TCMR declined (11% and 5% at follow-up, respectively), whereas the percentage of molecular AMR increased to 31% (all Table 1). Overall, 55% of baseline biopsies and 60% of follow-up biopsies with histological rejection or subthreshold histological rejection lesions were molecularly confirmed. The molecular confirmation rate at baseline varied across histological subgroups, ranging from 27% in probable AMR to 69% in AMR cases. Among biopsies with histological TCMR, a greater number were classified as mixed molecular AMR/TCMR rather than molecular TCMR alone; some of these showed evidence of MVI (all Supplementary Table S3).

### Molecular Differentiation Among Histologically “Rejection-Free” Baseline Biopsies

**Figure 1:**
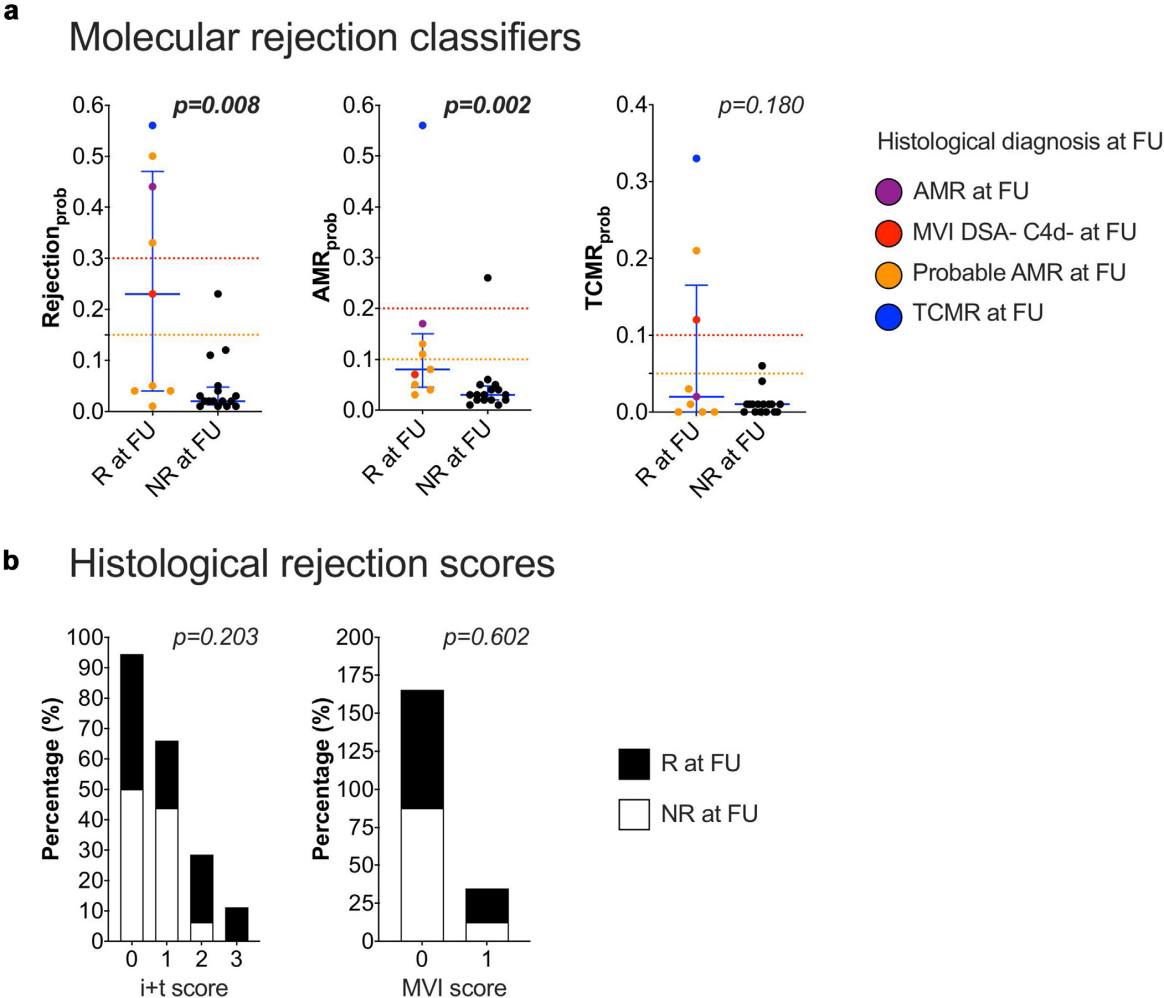
Suspicious molecular signals in baseline biopsies without histological rejection. Twenty-five baseline biopsies without histological rejection are categorized into biopsies with histological rejection (R at FU; *n* = 9) and without histological rejection at follow-up (NR at FU; *n* = 16). (a) Molecular rejection classifiers, compared using Mann Whitney U-test. The dashed lines indicate subthreshold (orange) and threshold levels (red) for each molecular score. (b) Histological i + t and MVI scores between the 2 groups, compared using chi-square or Fisher exact test, as appropriate. FU, follow-up; MVI, microvascular inflammation.

To evaluate the added value of MMDx in detecting early or subthreshold molecular rejection signals, we investigated the 25 of 80 (31%) biopsy series that showed no histological rejection signs at baseline. Nine of 25 (36%) developed histological rejection at follow-up (1 case with AMR; 1 case with MVI, DSA-negative, and C4d-negative; 1 case with TCMR, and 6 cases with probable AMR; Figure 1). In 5 of 9 baseline cases (56%), Rejection_prob_ was at least >0.15 (subthreshold cutoff), in 4 of 9 (44%), AMR_prob_ was >0.1, and in 3 of 9 (33%) TCMR_prob_ was >0.05. In baseline biopsies without histological rejection at follow-up, only 1 of 16 (6%) demonstrated relevant molecular rejection activity. Median (interquartile range) Rejection_prob_ was 0.23 (0.04–0.47) in baseline biopsies with histological rejection at follow-up, compared with 0.02 (0.01–0.05) in cases without histological rejection at follow-up (*P* = 0.008). In addition, median AMR_prob_ was 0.08 (0.05–0.15) compared with 0.03 (0.02–0.05) (*P* = 0.002; Figure 1 and Table 2). In addition, specific molecular lesion scores, as well as all AMR archetype scores (R7; median 0.30 vs. 0.09, *P* = 0.024) differed significantly between the 2 investigated subgroups. Histologically, there was no significant difference in either the i + t (inflammation) score or the g + ptc (MVI) score between the subgroups (*P* = 0.411 and *P* = 0.602, respectively; Table 2).

**Table 2:** Differentiation of baseline biopsies without histological rejection

	Histological rejection at follow-up (*n* = 9)	No histological rejection at follow-up (*n* = 16)	*P*
Histological lesions			
Inflammation (I + t), mean (SD)	1 ± 1.12	0.56 ± 0.63	0.411
MVI (g + ptc), mean (SD)	0.22 ± 0.44	0.13 ± 0.34	0.602
Interstitial inflammation (i), mean (SD)	0	0	NA
Tubulitis (t), mean (SD)	1 ± 1.12	0.56 ± 0.63	0.411
Arteritis (v), mean (SD)	0.13 ± 0.35	0.25 ± 0.45	0.631
Glomerulitis (g), mean (SD)	0.22 ± 0.44	0.13 ± 0.34	0.602
Peritubular capillaritis (ptc), mean (SD)	0	0	NA
Molecular classifier scores			
Injury scores			
GD score, median (IQR)	−0.59 (−2.90 to 2.45)	−2.06 (−3.30 to −0.37)	0.276
AKI score, median (IQR)	0.10 (−0.61 to 0.82)	0 (−0.47 to 0.43)	0.879
IFTA score, median (IQR)	0.09 (0.07–0.82)	0.20 (0.08–0.42)	0.728
Rejection scores			
Rejection_prob_, median (IQR)	0.23 (0.04–0.47)	0.02 (0.01–0.05)	0.008
AMR_prob_, median (IQR)	0.08 (0.05–0.15)	0.03 (0.02–0.05)	0.002
TCMR_prob_, median (IQR)	0.02 (0–0.17)	0.01 (0–0.01)	0.180
Lesion scores			
g_prob_, median (IQR)	0.17 (0.10–0.37)	0.11 (0.07–0.15)	0.030
ptc_prob_, median (IQR)	0.17 (0.09–0.41)	0.07 (0.05–0.08)	0.003
i_prob_, median (IQR)	0.03 (0.02–0.24)	0.02 (0.01–0.03)	0.117
t_prob_, median (IQR)	0.07 (0.03–0.30)	0.03 (0.02–0.04)	0.062
Archetype scores			
R1 – non-rejecting, median (IQR)	0.71 (0.33–0.88)	0.91 (0.86–0.94)	0.017
R2 – TCMR2, median (IQR)	0 (0–0.09)	0 (0)	0.091
R3 – TCMR1, median (IQR)	0 (0–0.01)	0 (0)	0.447
R4 – early-stage AMR, median (IQR)	0.09 (0.05–0.35)	0.05 (0.02–0.08)	0.075
R5 – fully-developed AMR, median (IQR)	0 (0–0.07)	0 (0–0.01)	0.418
R6 – late-stage AMR, median (IQR)	0.02 (0–0.19)	0.03 (0–0.08)	0.944
R7 (all AMR), median (IQR)	0.30 (0.10–0.42)	0.09 (0.06–0.15)	0.024
R8 (all TCMR), median (IQR)	0.01 (0–0.23)	0 (0)	0.042

AKI, acute kidney injury; AMR, antibody-mediated rejection; GD, global disturbance; IFTA, atrophy-fibrosis; IQR, Interquartile range; prob, probability; MVI, microvascular inflammation; TCMR, T-cell–mediated rejection.

Continuous variables are reported as median and IQR or mean and SD. Categorical variables as numbers (*n*) and percentages (%). Injury scores are compared regardless of the possibility of different training sets. R7 = R4 + R5 + R6 (all AMR score), whereas R8 = R2 + R3 (all TCMR score). Mann Whitney U-test is used for comparing of the subgroups.

### Antirejection Treatment Responses in Molecular Rejection Scenarios

**Figure 2:**
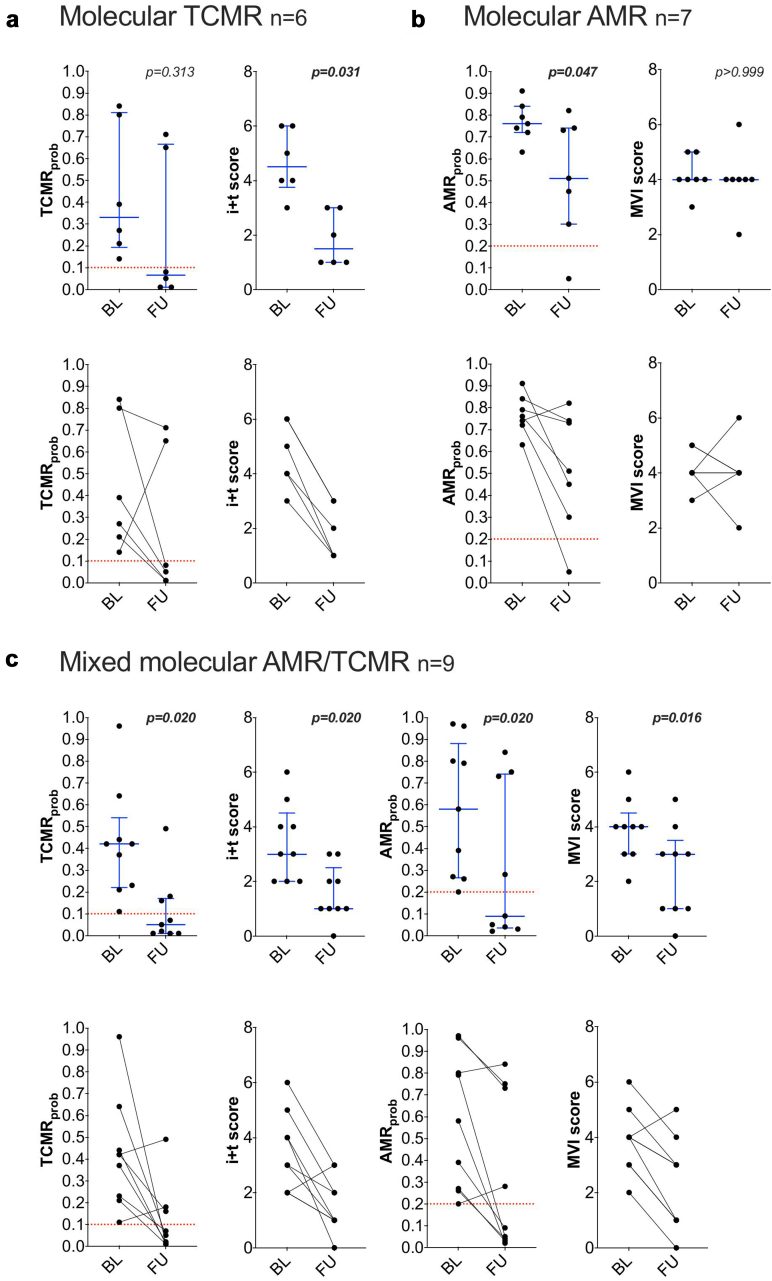
Conventional antirejection treatments among molecular rejection subgroups. Baseline (BL) and follow-up (FU) biopsies are compared for each scenario using Wilcoxon signed rank-test. The red dashed lines indicate thresholds levels for each molecular score. Median and interquartile range are demonstrated in blue and the course of each biopsy series below as connected dots. (a) Molecular TCMR cases receiving conventional treatment (*n* = 6). TCMR classifier scores and histological i + t scores are demonstrated. (b) Molecular AMR cases receiving conventional treatment (*n* = 7). AMR classifier scores and histological MVI scores are presented. (c) Mixed molecular AMR/TCMR cases receiving treatment (*n* = 9). All relevant molecular rejection and histological scores are visualized. AMR, antibody-mediated rejection; MVI, microvascular inflammation; TCMR, T-cell–mediated rejection.

In Table 3, we outline the conventional antirejection therapies used in the 3 main treatment scenarios for molecular rejection as follows: molecular TCMR (*n* = 6), molecular AMR (*n* = 7), and mixed molecular AMR/TCMR (*n* = 9). Among cases with molecular TCMR, 4 of 6 (67%) demonstrated a significant reduction of TCMR_prob_ to < 0.1 following treatment. Overall, there was no significant effect of reduction in median TCMR_prob_ (*P* = 0.313), driven by the 2 of 6 cases (33%) remaining at high TCMR_prob_ levels (progressing to mixed phenotypes). However, histological i + t scores showed a significant reduction at follow-up (median 4.5 to 1.5; mean ± SD from 4.67 ± 1.11 to 1.83 ± 0.90; *P* = 0.031; Table 4 and Figure 2). Among cases with molecular AMR receiving conventional antirejection treatment, only 1 of 7 cases (14%) showed a significant reduction in AMR_prob_ to < 0.2 at follow-up, whereas 6 of 7 cases (86%) remained above the cutoff. However, within the entire subgroup, AMR_prob_ significantly decreased (median 0.76 to 0.51; *P* = 0.047; Table 4 and Figure 2). In contrast, histological MVI scores remained unchanged, with a persistent median of g + ptc = 4 (*P* > 0.99), and none of the 7 cases had an MVI score < 2 at follow-up, as shown in Figure 2. Furthermore, 5 of 7 (71%) within this subgroup presented histological signs of chronic-active AMR (either glomerular basement membrane double contours (i.e., cg) > 0 or peritubular capillary basement membrane multilayering (i.e., ptcml) 1). In the subgroup with mixed molecular AMR/TCMR, we observed significant reductions in both AMR_prob_ (median: 0.58 to 0.09; *P* = 0.02) and TCMR_prob_ (median 0.42 to 0.05; *P* = 0.02), as well as histological i + t scores (mean ± SD from 3.44 ± 1.34 to 1.56 ± 0.96; *P* = 0.02) and MVI scores (mean ± SD from 3.89 ± 1.10 to 2.33 ± 1.56; *P* = 0.016), as demonstrated in Table 4 and Figure 2. Only 3 of 9 cases (33%) demonstrated histological signs of chronic-active AMR. In addition, of the 9 cases, 5 (56%) demonstrated resolution of molecular AMR rejection activity at follow-up (AMR_prob_ < 0.2). Among these, 4 of 5 cases (80%) also showed a concomitant reduction in MVI scores to below threshold levels (g + ptc < 2). Notably, in a subgroup of molecular AMR cases with late AMR features treated with antiinterleukin 6 therapy (*n* = 10; also listed in Table 3), no changes were observed in molecular classifier scores or histological findings at follow-up (Table 4 and Supplementary Figure S2). The histological disease course for each of the 3 main scenarios is summarized in Supplementary Table S4, whereas the histological disease course for cases without molecular rejection activity at baseline receiving treatment (*n* = 15) is demonstrated in Supplementary Table S5. Five cases with molecular rejection were not treated during the biopsy series (2 molecular AMR, 1 mixed molecular AMR/TCMR, and 2 molecular TCMR). In 2 of these cases, treatment was initiated immediately after the second biopsy because of persistent molecular rejection; 1 patient with AMR was converted to belatacept. The remaining 2 cases showed no clear histological signs of rejection.

**Table 3:** Different scenarios of molecular rejection with conventional antirejection treatments and molecular AMR scenario with antiinterleukin 6 treatment

	All patients (*N* = 80)
**Scenario 1:** Molecular TCMR receiving conventional antirejection therapy, n (%)	6 (8%)
Pulsed steroids, *n* (%)	6 (100%)
ATG, *n* (%)	2 (33%)
PLEX/i.v. Ig, *n* (%)	3 (50%)
**Scenario 2:** Molecular AMR receiving conventional antirejection therapy, *n* (%)	7 (9%)
Pulsed steroids, *n* (%)	7 (100%)
PLEX/i.v. Ig, *n* (%)	5 (71%)
RTX, n (%)	3 (43%)
**Scenario 3:** Mixed molecular AMR/TCMR receiving conventional antirejection therapy, *n* (%)	9 (11%)
Pulsed steroids, *n* (%)	9 (100%)
ATG, *n* (%)	4 (44%)
PLEX/i.v. Ig, *n* (%)	8 (89%)
RTX, *n* (%)	5 (56%)
**Scenario 4:** Molecular AMR receiving antiinterleukin-6–directed therapy, *n* (%)	10 (13%)
Tocilizumab, *n* (%)	7 (70%)
Clazakizumab, *n* (%)	3 (30%)

AMR, antibody-mediated rejection; ATG, antithymocyte globulin; PLEX, plasmapheresis; RTX, rituximab; TCMR, T-cell–mediated rejection.

Categorical variables are presented as numbers (*n*) and percentages (%) of total (for bold values) or percentages (%) of the corresponding subgroup (for nonhighlighted values).

**Table 4:** Antirejection treatment responses based on histology and MMDx

	Scenario 1 (*n* = 6)			Scenario 2 (*n* = 7)			Scenario 3 (*n* = 9)			Scenario 4 (*n* = 10)		
	BL	FU	*P*	BL	FU	*P*	BL	FU	*P*	BL	FU	*P*
Histological lesions												
Inflammation (I + t), mean (SD)	4.67 ± 1.11	1.83 ± 0.90	0.031	1.29 ± 0.88	1 ± 0.93	0.500	3.44 ± 1.34	1.56 ± 0.96	0.020	1.10 ± 0.54	1.10 ± 0.30	>0.99
MVI (g + ptc), mean (SD)	2.33 ± 1.80	1.33 ± 1.11	0.375	4.14 ± 0.64	4 ± 1.07	>0.99	3.89 ± 1.10	2.33 ± 1.56	0.016	2.90 ± 1.22	3.30 ± 1	0.406
MVI at/above threshold, n (%)	4 (67)	3 (50)	1	7 (100)	7 (100)	1	9 (100)	5 (56)	0.082	8 (80)	10 (100)	0.474
Arteritis (v), mean (SD)	0.80 ± 0.75	0.33 ± 0.47	0.625	0.57 ± 0.49	0.43 ± 0.49	>0.99	1.25 ± 0.97	0.67 ± 0.67	0.344	0.11 ± 0.31	0.30 ± 0.46	0.500
Arteriolar hyalinosis (ah), mean (SD)	0.67 ± 0.47	1 ± 0.58	0.500	1.43 ± 0.90	1.29 ± 1.03	>0.99	0.89 ± 0.87	1.11 ± 0.87	0.625	2.20 ± 0.60	2.50 ± 0.67	0.250
Molecular classifier scores												
Injury scores												
GD score, median (IQR)	4.46 (2.16–5.18)	−0.10 (−0.87 to 3.00)	0.219	1.26 (0.26–1.76)	0.57 (−0.55 to 2.60)	0.844	4.73 (4.45–6.17)	1.21 (−2.84 to 3.15)	0.027	0.77 (−0.37 to 1.52)	0.47 (−1.09 to 1.40)	0.922
AKI score, median (IQR)	1.39 (0.75–1.64)	0.37 (0.20–0.74)	0.063	0.14 (0.01–0.26)	0.36 (−0.03 to 0.67)	0.609	1.13 (0.93–1.49)	0.23 (0.06–0.89)	0.027	−0.09 (−0.38 to 0.41)	0.06 (−0.11 to 0.41)	0.744
IFTA score, median (IQR)	0.41 (0.26–0.55)	0.62 (0.43–0.78)	0.156	0.22 (0.15–0.34)	0.60 (0.29–0.81)	0.078	0.61 (0.55–0.66)	0.38 (0.12–0.79)	0.602	0.48 (0.24–0.81)	0.42 (0.29–0.55)	0.625
Rejection scores												
Rejection_prob_, median (IQR)	0.60 (0.46–0.78)	0.23 (0.04–0.66)	0.094	0.86 (0.79–0.89)	0.69 (0.56–0.75)	0.031	0.95 (0.90–0.95)	0.61 (0.15–0.69)	0.012	0.71 (0.61–0.82)	0.65 (0.57–0.69)	0.752
AMR_prob_, median (IQR)	0.12 (0.10–0.13)	0.07 (0.04–0.30)	0.844	0.76 (0.73–0.82)	0.51 (0.38–0.74)	0.047	0.58 (0.27–0.80)	0.09 (0.04–0.73)	0.020	0.54 (0.33–0.72)	0.63 (0.44–0.69)	0.375
TCMR_prob_, median (IQR)	0.33 (0.23–0.70)	0.07 (0.02–0.51)	0.313	0.02 (0.01–0.04)	0.01 (0.01–0.03)	0.875	0.42 (0.23–0.44)	0.05 (0.01–0.16)	0.020	0.02 (0.02–0.04)	0.02 (0.01–0.02)	0.438
Lesion scores												
g_prob_, median (IQR)	0.28 (0.25–0.33)	0.15 (0.12–0.25)	0.188	0.73 (0.71–0.84)	0.67 (0.49–0.70)	0.094	0.63 (0.55–0.85)	0.22 (0.13–0.67)	0.008	0.63 (0.58–0.72)	0.62 (0.41–0.70)	0.721
ptc_prob_, median (IQR)	0.53 (0.51–0.53)	0.21 (0.14–0.53)	0.156	0.82 (0.78–0.83)	0.71 (0.62–0.77)	0.016	0.83 (0.80–0.92)	0.55 (0.22–0.71)	0.004	0.72 (0.61–0.79)	0.66 (0.58–0.71)	0.826
i_prob_, median (IQR)	0.66 (0.35–0.90)	0.07 (0.02–0.41)	0.156	0.07 (0.05–0.10)	0.03 (0.03–0.12)	0.438	0.74 (0.53–0.92)	0.15 (0.02–0.46)	0.016	0.06 (0.04–0.10)	0.04 (0.02–0.09)	0.787
t_prob_, median (IQR)	0.40 (0.32–0.78)	0.13 (0.08–0.57)	0.313	0.06 (0.06–0.11)	0.07 (0.05–0.11)	0.813	0.56 (0.45–0.59)	0.15 (0.05–0.27)	0.012	0.08 (0.05–0.13)	0.05 (0.04–0.09)	0.484
ah_prob_, median (IQR)	0.23 (0.16–0.24)	0.40 (0.37–0.51)	0.063	0.48 (0.31–0.61)	0.71 (0.68–0.72)	0.063	0.27 (0.22–0.33)	0.50 (0.41–0.59)	0.031	0.75 (0.66–0.82)	0.68 (0.58–0.77)	>0.99
Archetype scores												
R1 – non-rejecting, median (IQR)	0.07 (0–0.19)	0.64 (0.15–0.71)	0.125	0 (0–0.01)	0 (0–0.09)	0.250	0 (0)	0.13 (0.03–0.72)	0.016	0 (0–0.14)	0.10 (0–0.26)	0.469
R2 – TCMR2, median (IQR)	0.52 (0.35–0.76)	0.09 (0.02–0.14)	0.063	0 (0)	0 (0–0.02)	NA	0.17 (0–0.41)	0.02 (0–0.14)	0.211	0.01 (0–0.03)	0 (0)	0.125
R3 – TCMR1, median (IQR)	0 (0–0.13)	0 (0–0.16)	0.750	0 (0–0.01)	0 (0–0.03)	>0.99	0.43 (0.15–0.56)	0 (0)	0.039	0 (0–0.01)	0 (0–0.02)	0.625
R4 – early-stage AMR, median (IQR)	0.23 (0.06–0.25)	0.09 (0.02–0.11)	0.313	0.39 (0.01–0.65)	0.45 (0.21–0.53)	>0.99	0.03 (0–0.21)	0.18 (0.12–0.41)	0.344	0.53 (0.47–0.61)	0.25 (0.11–0.48)	0.084
R5 – fully-developed AMR, median (IQR)	0 (0)	0 (0)	>0.99	0.49 (0.33–0.77)	0.26 (0.11–0.43)	0.078	0.14 (0–0.44)	0 (0–0.05)	0.063	0.27 (0.14–0.35)	0.37 (0.29–0.56)	0.106
R6 – late-stage AMR, median (IQR)	0.03 (0–0.14)	0.14 (0.02–0.28)	0.625	0 (0–0.12)	0.12 (0.03–0.25)	0.313	0 (0–0.01)	0.06 (0–0.16)	0.031	0.01 (0–0.14)	0.03 (0–0.34)	0.125
R7 (all AMR), median (IQR)	0.31 (0.06–0.46)	0.27 (0.16–0.32)	0.625	0.99 (0.96–1)	0.93 (0.84–0.98)	0.125	0.44 (0.32–0.57)	0.52 (0.25–0.69)	0.820	0.92 (0.84–1)	0.82 (0.75–0.97)	0.432
R8 (all TCMR), median (IQR)	0.52 (0.35–0.91)	0.09 (0.02–0.49)	0.219	0 (0–0.04)	0 (0–0.05)	0.750	0.56 (0.43–0.68)	0.14 (0.02–0.30)	0.020	0.01 (0–0.07)	0 (0–0.02)	0.734

AKI, acute kidney injury; AMR, antibody-mediated rejection; GD, global disturbance; IFTA, atrophy-fibrosis; IQR, Interquartile range; MVI, microvascular inflammation; TCMR, T-cell–mediated rejection.

Continuous variables are reported as median and IQR or mean and SD; categorical variables as numbers (*n*) and percentages (%). Scenarios 1 to 4 refer to the descriptions in Table 3. Baseline (BL) with molecular rejection and corresponding follow-up (FU) biopsies are compared. Wilcoxon signed rank- or Fisher exact test were used to compare BL and FU biopsies.

### Gradual Rejection Phenotyping and Disease Course of Molecular Rejection

**Figure 3:**
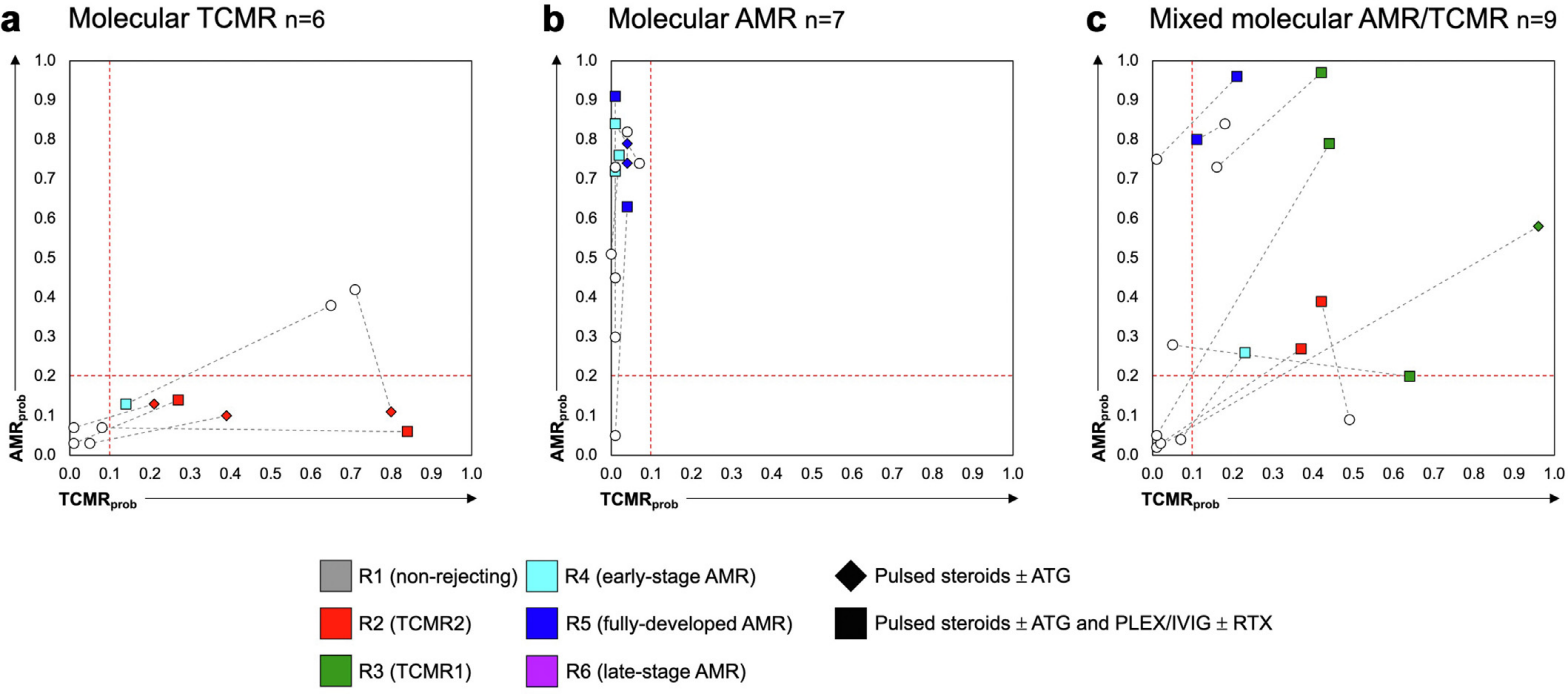
Gradual disease course in different molecular rejection settings. Scatter plots with AMR_prob_ (y-axis) and TCMR_prob_ are demonstrated for each molecular rejection setting receiving conventional antirejection treatment: (a) Molecular TCMR cases receiving conventional treatment (*n* = 6). (b) Molecular AMR cases receiving conventional treatment (*n* = 7). (c) Mixed molecular AMR/TCMR cases receiving treatment (*n* = 9). The red dashed lines indicate the threshold levels for each score. Baseline biopsies are categorized by color (predominant archetype) and shape (type of antirejection treatment), corresponding follow-up biopsies are visualized as white dots. AMR, antibody-mediated rejection; TCMR, T-cell–mediated rejection.

In Figure 3, we illustrate the disease course in the 3 main molecular treatment scenarios: (i) molecular TCMR, (ii) molecular AMR, and (iii) mixed molecular AMR/TCMR, all treated with conventional antirejection therapy. In molecular TCMR cases, 4 of 6 cases (67%) showed complete resolution of molecular rejection activity (AMR_prob_ < 0.2 and TCMR_prob_ < 0.1), whereas the remaining cases progressed to a mixed molecular AMR/TCMR phenotype (Figure 3a). Notably, 1 case exhibited a predominant R4 (early-stage AMR) phenotype, indicating significant AMR activity at baseline, whereas all other cases were classified primarily as R2 (TCMR2). Among molecular AMR cases treated with conventional therapy, 6 of 7 (cases 86%) retained a molecular AMR phenotype after treatment, whereas only 1 case (14%) showed resolution of rejection activity (Figure 3b). No cases transitioned to a molecular TCMR or mixed molecular AMR/TCMR phenotype. All cases exhibited either a predominant R4 (early-stage AMR) or R5 (fully-developed AMR) archetype. In the subgroup with mixed molecular AMR/TCMR treated with conventional antirejection therapy (Figure 3c), rejection progressed in the following 3 possible directions: (i) toward molecular AMR, observed in cases with predominant R3 (TCMR1, formerly mixed) or R5 (fully-developed AMR) archetypes; toward molecular TCMR, as seen in a single case with an R2 archetype at baseline; or (iii) toward complete resolution of molecular rejection activity (cases with predominant R2, R3, or R4 archetypes). Plotting baseline biopsies (*n* = 80) based on their histological diagnosis against AMR_prob_ and TCMR_prob_ revealed a heterogeneous distribution (Supplementary Figure S3). The disease course of molecular negative cases receiving treatment demonstrated 7 of 15 cases (47%) progressing to molecular rejection at follow-up, all of them having some sort of histological rejection at baseline (Supplementary Figure S4).

## Discussion

Biopsy-based transcriptomics provide deeper insights into kidney allograft rejection phenotypes than traditional histopathology alone, leveraging big data and machine learning. However, validation across diverse cohorts is essential. In this study, we evaluated MMDx as a follow-up tool in a well-characterized transplant cohort, demonstrating its ability to detect molecular rejection signals among histologically “rejection-free” biopsies, monitor treatment responses in addition to or beyond visible changes in histology, and track disease progression along the molecular AMR and TCMR spectrum.

We observed that baseline biopsies without histological rejection at baseline but histological rejection at follow-up (*n* = 9) have higher molecular activity across all rejection classifiers (Rejection_prob_, AMR_prob_, and TCMR_prob_), as well as archetype scores (especially “all AMR”, namely R4 + R5 + R6), compared with “rejection-free” baseline biopsies without rejection at follow-up. Notably, only approximately one-third to 50% of cases with subsequent rejection had relevant rejection activity at approximately or above validated cutoffs from the MMDx platform. This implies that nearly 50% of cases eventually developing histological rejection had subthreshold molecular findings, calling into question the sensitivity of these thresholds. This aligns with observations by Halloran *et al.*, who highlighted the importance of subthreshold molecular activity. Their study found that many histologically “rejection-free” biopsies showed subthreshold molecular activity for TCMR and AMR, which was linked to later rejection.^14^ Therefore, subthreshold molecular signals may play a critical role in the early identification of rejection activity, suggesting a need to reassess diagnostic thresholds or consider early treatment in critical cases. In addition, in baseline biopsies diagnosed as probable AMR by histology (*n* = 11), MMDx detected subthreshold AMR_prob_ (> 0.1) in 3 of the 5 cases that later progressed to histological rejection, compared with only 1 of the 6 cases that remained probable AMR or showed resolution (data not shown). This suggests that MMDx may help further distinguish this high-risk subgroup.^16^ In summary, these findings indicate that cases with subthreshold molecular lesions or histological indicators of AMR (such as probable AMR) could potentially benefit from early therapeutic consideration. Conversely, cases without signs suspicious of rejection might be more suitable for continued monitoring through follow-up biopsies.

Once rejection has developed, clinicians often initiate conventional antirejection treatments such as pulsed steroids or more intensive protocols that include antithymocyte globulin, plasmapheresis/i.v. Ig, or rituximab. However, histological assessments may not always capture the full impact of these treatments, as shown in previous studies.^21^^,^^22^ In contrast, MMDx may have the potential to more accurately measure treatment responses through its continuous classifiers for AMR and TCMR. In our study, we categorized conventional antirejection treatments into the following 3 groups: (i) molecular TCMR receiving treatment; (ii) molecular AMR receiving antirejection therapy; and (iii) mixed molecular AMR/TCMR undergoing antirejection treatment. In cases with treated molecular TCMR (scenario 1), reductions were observed not only in histological inflammation and tubulitis (i and t lesions) but also in molecular scores, including TCMR classifier scores, where 67% of cases showed complete resolution of TCMR_prob_ activity after treatment. Notably, AKI scores showed a trend toward a significant reduction in the subgroup (*P* = 0.063). Because AKI scores, reflecting injury-repair response-associated transcripts, have been linked to clinical outcomes, this reduction suggests a favorable prognosis following effective TCMR therapy.^23^ Similar observations were made in the subgroup of mixed molecular AMR/TCMR receiving treatment. All histological parameters and classifier scores showed a significant decline at follow-up, including AKI scores. However, in pure molecular AMR cases treated for rejection, no significant changes in histological parameters were observed. Even though molecular analysis revealed overall reductions in rejection and AMR classifier scores, only 1 case showed resolution of AMR classifier activity, whereas 6 out of 7 cases remained above upper limits of normal. In addition, unlike scenario 1 and 3 (TCMR and mixed), molecular injury scores, especially AKI scores, showed no significant improvement, indicating a more complex injury pattern in AMR cases or a later stage in the disease course of rejection. A recent study exploring the CD38-targeting antibody, felzartamab showed promising reductions in histological MVI, as well as molecular rejection scores and injury scores. This was accompanied by reductions in biomarkers such as donor-derived cell-free DNA and natural killer cell counts. These findings raise several questions about the efficacy of current AMR treatments (steroids, plasmapheresis/i.v. Ig, rituximab).^24^ Are these therapies generally ineffective, or do only very limited and early AMR cases benefit, necessitating molecular differentiation? Potentially, the complex pathophysiology of MVI, even more if combined with signs of chronic-active injury (cg/ptcml), requires more targeted interventions, such as felzartamab, which addresses both plasma cells and natural killer cells. Interestingly, in a subgroup of molecular patients with AMR treated with tocilizumab or clazakizumab within our cohort, we observed no histological or molecular responses, casting doubt on the effectiveness of interleukin-6–targeted therapy. The absence of an effect on molecular rejection scores has previously been demonstrated in a phase 2 trial evaluating clazakizumab.^10^

Notably, in our cohort, cases with mixed molecular AMR/TCMR appeared to respond better to conventional treatments, suggesting that these may represent “earlier” or “distinct” rejection phenotypes, compared with our subgroup of molecular AMR. This group showed fewer features indicative of a chronic-active AMR component. This was further demonstrated by their disease course: cases with predominant R2 to R4 archetypes often responded to treatment, whereas cases with predominant R5 archetypes tended to maintain high AMR classifier activity. Among cases with molecular TCMR that received treatment, the disease either showed a tendency to complete resolution of rejection activity or progression into a mixed molecular AMR/TCMR phenotype, but did not transition directly to molecular AMR. In contrast, cases with molecular AMR tended to remain within their rejection phenotype.

This again highlights that molecular TCMR and mixed molecular AMR/TCMR might be more dynamic and responsive phenotypes, emphasizing adequate TCMR treatment, whereas molecular AMR represents a more advanced and complex rejection state, presumably needing more advanced therapies. This is further supported by the observation that molecular TCMR and mixed molecular AMR/TCMR were more common at baseline, whereas molecular AMR cases were more frequent at follow-up, indicating an overall disease progression within the cohort (Table 1). In conclusion, these 3 scenarios illustrate a dynamic transition between different molecular disease states, as reflected by molecular classifiers—from no molecular rejection to molecular TCMR, to mixed molecular AMR/TCMR—with the potential for either resolution or progression to molecular AMR. This gradual categorization is not well-captured by histology, because many cases diagnosed as “no rejection” by histology still exhibit molecular rejection, and *vice versa* (Supplementary Figure S3).

Despite the significance of our findings, several limitations must be acknowledged. The relatively small sample size restricts the generalizability of our conclusions, which should be considered primarily hypothesis-generating. In addition, there is a potential selection bias, because many biopsies were performed because of DSA positivity and were “subclinical” in nature. Another limitation is the heterogeneity of the cohort and the absence of prespecified outcomes, because treatment decisions were made on an individual case basis. Nevertheless, this study provides valuable insights from an independent, real-world clinical cohort and is among the first to evaluate the longitudinal use of MMDx across a series of biopsies.

## Conclusion

Our study suggests that MMDx could be useful for detecting suspicious subthreshold rejection activity in early disease stages when histological findings are ambiguous or absent. This applies to both molecular classifier and archetype scores and could prompt clinicians to initiate treatment at an early stage or to perform follow-up biopsies. Furthermore, MMDx can be used as a monitoring tool to assess continuous molecular classifier scores and detect treatment responses beyond what is visible through histology, especially in cases receiving intensified treatments. Ultimately, MMDx enables a more nuanced and gradual phenotyping of rejection, compared with the binary approach of histology. This is evident in the analysis of follow-up biopsies, which illustrate various patterns of resolution or progression within the AMR and TCMR continuum; in some cases depending on the predominant archetype clusters. This could help determine which patient groups might benefit the most from targeted antirejection interventions.

## Disclosure

All the authors declared no competing interests.

## Supplementary Material

Supplementary File (PDF) **Figure S1.** Deduction of the study cohort. **Figure S2.** Antiinterleukin 6 antirejection treatment among molecular AMR. **Figure S3.** Distribution of histological baseline diagnoses along AMR and TCMR classifier activity. **Figure S4.** Gradual disease course in molecular negative cases receiving antirejection treatment. **Table S1.** Additional basic and immunological characteristics. **Table S2.** Basic, biopsy and follow-up characteristics of histological subgroups at baseline. **Table S3.** Confirmation of histology by MMDx. **Table S4.** Histological disease course in molecular rejection scenarios. **Table S5.** Histological disease course in molecular negative cases receiving antirejection treatment. STROBE Checklist.

## Footnotes

Supplementary File (PDF)
**Figure S1.** Deduction of the study cohort. **Figure S2.** Antiinterleukin 6 antirejection treatment among molecular AMR. **Figure S3.** Distribution of histological baseline diagnoses along AMR and TCMR classifier activity. **Figure S4.** Gradual disease course in molecular negative cases receiving antirejection treatment. **Table S1.** Additional basic and immunological characteristics. **Table S2.** Basic, biopsy and follow-up characteristics of histological subgroups at baseline. **Table S3.** Confirmation of histology by MMDx. **Table S4**. Histological disease course in molecular rejection scenarios. **Table S5.** Histological disease course in molecular negative cases receiving antirejection treatment. STROBE Checklist.
