# Supplementary material for: Biopsy-Based Transcriptomics Support Rejection Monitoring Through Repeated Kidney Allograft Biopsies
Source: Kidney Int Rep. 2025 Apr 27;10(7):2357–68. doi: 10.1016/j.ekir.2025.04.043 (PMC12266197; doi:10.1016/j.ekir.2025.04.043)
Supplement: Supplementary File (PDF) — Figure S1. Deduction of the study cohort. Figure S2. Antiinterleukin 6 antirejection treatment among molecular AMR. Figure S3. Distribution of histological baseline diagnoses along AMR and TCMR classifier activity. Figure S4. Gradual disease course in molecular negative cases receiving antirejection treatment. Table S1. Additional basic and immunological characteristics. Table S2. Basic, biopsy and follow-up characteristics of histological subgroups at baseline. Table S3. Confirmation of histology by MMDx. Table S4. Histological disease course in molecular rejection scenarios. Table S5. Histological disease course in molecular negative cases receiving antirejection treatment. STROBE Checklist. [file mmc1.pdf]

SUPPLEMENTARY MATERIALS

"Biopsy-based Transcriptomics Support Rejection Monitoring through Repeated Kidney Allograft Biopsies"

	All patients (n=80)
**Maintenance IS at baseline bx**	
Tacrolimus, n (%)	67 (84)
Cyclosporine, n (%)	11 (14)
Sirolimus/Everolimus, n (%)	4 (5)
Belatacept, n (%)	0 (0)
Mycophenolate, n (%)	69 (86)
Azathioprine, n (%)	6 (8)
Prednisone, n (%)	62 (78)
**Maintenance IS at follow-up bx**	
Tacrolimus, n (%)	67 (84)
Cyclosporine, n (%)	4 (5)
Sirolimus/Everolimus, n (%)	4 (5)
Belatacept, n (%)	8 (10)
Mycophenolate, n (%)	70 (88)
Azathioprine, n (%)	4 (5)
Prednisone, n (%)	73 (91)
**HLA mismatches in detail**	
HLA-A	
0, n (%)	12 (15)
1, n (%)	36 (45)
2, n (%)	32 (40)
HLA-B	
0, n (%)	8 (10)
1, n (%)	26 (33)
2, n (%)	46 (58)
HLA-DRB	
0, n (%)	10 (13)
1, n (%)	48 (60)
2, n (%)	22 (28)
**Type of TPL**	
ABOc, n (%)	29 (36)
ABOi, n (%)	9 (11)
DBD, n (%)	29 (36)
DCD, n (%)	9 (11)
SPK, n (%)	4 (5)
**Underlying disease type**	
Hypertensive nephropathy, n (%)	4 (5)
Diabetic nephropathy, n (%)	8 (10)
ADPKD, n (%)	14 (18)
CAKUT, n (%)	3 (4)
GN/Vasculitis, n (%)	25 (31)
Other, n (%)	14 (18)
Unknown, n (%)	12 (15)

Supplementary Table S1. Additional basic and immunological characteristics.

Categorical variables are presented as numbers (n) and percentages (%).

	Mixed AMR/TCMR (n=3)	AMR (n=16)	MVI, DSA-negative and C4d-negative (n=11)	TCMR (n=11)	Borderline TCMR (n=3)	Probable AMR (n=11)	No histological rejection (n=25)
**Female sex, n (%)**	1 (33)	2 (13)	3 (27)	3 (27)	1 (33)	6 (55)	11 (44)
**Age at TPL (years), median (IQR)**	27 (12, 55)	29 (26, 43)	47 (39, 59)	48 (39, 54)	27 (15, 64)	45 (38, 49)	50 (41, 56)
**Living donation, n (%)**	1 (33)	9 (56)	6 (55)	5 (45)	1 (33)	6 (55)	10 (40)
**Repeat TPL, n (%)**	0 (0)	1 (6)	1 (9)	1 (9)	0 (0)	0 (0)	3 (12)
**Time between TPL and baseline bx (months), median (IQR)**	41 (8, 96)	98 (47, 140)	4 (3, 81)	8 (2, 35)	20 (10, 24)	41 (24, 129)	4 (1, 15)
**Time between baseline and follow-up bx (months), median (IQR)**	4 (1, 6)	16 (5, 19)	8 (6, 11)	9 (4, 18)	12 (2, 18)	15 (10, 22)	10 (4, 19)
**HLA mismatches ≥5, n (%)**	2 (67)	5 (31)	4 (36)	4 (36)	1 (33)	5 (45)	12 (48)
**DSA-presence at bx**							
pDSA, n (%)	2 (67)	3 (19)	0 (0)	1 (9)	0 (0)	3 (27)	4 (16)
dnDSA, n (%)	3 (100)	15 (94)	0 (0)	1 (9)	1 (33)	8 (73)	1 (4)
**Bx indication***							
DSA, n (%)	3 (100)	16 (100)	0 (0)	2 (18)	1 (33)	11 (100)	4 (16)
eGFR decline, n (%)	2 (67)	7 (44)	7 (64)	10 (91)	1 (33)	2 (18)	13 (52)
Proteinuria rise, n (%)	0 (0)	1 (6)	5 (45)	3 (27)	1 (33)	2 (18)	8 (32)
Protocol bx, n (%)	0 (0)	0 (0)	2 (18)	0 (0)	0 (0)	0 (0)	5 (20)
**Histological subgroup at follow-up**							
Mixed AMR/TCMR, n (%)	0 (0)	0 (0)	0 (0)	0 (0)	1 (33)	0 (0)	0 (0)
AMR, n (%)	1 (33)	16 (100)	0 (0)	2 (18)	1 (33)	5 (45)	1 (4)
MVI, DSA-negative and C4d-negative, n (%)	0 (0)	0 (0)	7 (64)	1 (9)	0 (0)	0 (0)	1 (4)
TCMR, n (%)	2 (67)	0 (0)	0 (0)	3 (27)	1 (33)	0 (0)	1 (4)
Borderline TCMR changes, n (%)	0 (0)	0 (0)	0 (0)	0 (0)	0 (0)	0 (0)	0 (0)
Probable AMR, n (%)	0 (0)	0 (0)	0 (0)	0 (0)	0 (0)	4 (36)	6 (24)
No histological rejection, n (%)	0 (0)	0 (0)	4 (36)	5 (45)	0 (0)	2 (18)	16 (64)

Supplementary Table S2. Basic, biopsy and follow-up characteristics of histological subgroups at baseline. Continuous variables are reported as median and IQR. Categorical variables as numbers (n) and percentages (%). *One biopsy could have more than one indication.

		Corresponding molecular diagnosis				
	Total n=80	**Mixed molecular AMR/TCMR**	**Molecular AMR**	**Molecular TCMR**	**No molecular rejection**	**Confirmation (%)**
**Baseline biopsies** (Histological diagnosis)	**Mixed AMR/TCMR**	2	0	0	1	67%
	**AMR**	2	9	1	4	69%
	**MVI, DSA-, C4d-**	1	4	1	5	45%
	**Probable AMR**	0	3	0	8	27%
	**TCMR**	5	1	3	2	73%
	**Borderline changes**	0	0	1	2	33%
	**No histological rejection**	1	1	2	21	84%
	**Total:** Rejection confirmation in 30/55 (55%) / no molecular rejection confirmation in 21/25 (84%)					
	Total n=80	**Mixed molecular AMR/TCMR**	**Molecular AMR**	**Molecular TCMR**	**No molecular rejection**	**Confirmation (%)**
**Follow-up biopsies** (Histological diagnosis)	**Mixed AMR/TCMR**	1	0	0	0	100%
	**AMR**	4	15	1	6	73%
	**MVI, DSA-, C4d-**	1	7	1	0	100%
	**Probable AMR**	0	1	0	9	10%
	**TCMR**	3	1	1	2	57%
	**Borderline changes**	0	0	0	0	-
	**No histological rejection**	0	1	1	25	93%
	**Total:** Rejection confirmation in 33/55 (60%) / no molecular rejection confirmation in 25/27 (93%)					

Supplementary Table S3. Confirmation of histology by MMDx. Green indicates a confirmation of rejection and orange the opposite. Blue indicates a correct confirmation of the absence of rejection, whereas yellow indicates molecular rejection in the absence of histological rejection.

		(Follow-up)						
	Total n=6	**Mixed AMR/TCMR**	**AMR**	**MVI, DSA-, C4d-**	**Probable AMR**	**TCMR**	**Bordeline**	**No rejection**
**Scenario 1** (Baseline)	**Mixed AMR/TCMR**	0	0	0	0	0	0	0
	**AMR**	0	1	0	0	0	0	0
	**MVI, DSA-, C4d-**	0	0	0	0	0	0	1
	**Probable AMR**	0	0	0	0	0	0	0
	**TCMR**	0	2	0	0	0	0	1
	**Borderline**	0	0	0	0	0	0	0
	**No rejection**	0	0	0	1	0	0	0
	Total n=7	**Mixed AMR/TCMR**	**AMR**	**MVI, DSA-, C4d-**	**Probable AMR**	**TCMR**	**Bordeline**	**No rejection**
**Scenario 2** (Baseline)	**Mixed AMR/TCMR**	0	0	0	0	0	0	0
	**AMR**	0	3	0	0	0	0	0
	**MVI, DSA-, C4d-**	0	0	3	0	0	0	0
	**Probable AMR**	0	0	0	0	0	0	0
	**TCMR**	0	0	0	0	1	0	0
	**Borderline**	0	0	0	0	0	0	0
	**No rejection**	0	0	0	0	0	0	0
	Total n=9	**Mixed AMR/TCMR**	**AMR**	**MVI, DSA-, C4d-**	**Probable AMR**	**TCMR**	**Bordeline**	**No rejection**
**Scenario 3** (Baseline)	**Mixed AMR/TCMR**	0	1	0	0	1	0	0
	**AMR**	0	1	0	0	0	0	0
	**MVI, DSA-, C4d-**	0	0	1	0	0	0	0
	**Probable AMR**	0	0	0	0	0	0	0
	**TCMR**	0	0	1	0	2	0	2
	**Borderline**	0	0	0	0	0	0	0
	**No rejection**	0	0	0	0	0	0	0
	Total n=10	**Mixed AMR/TCMR**	**AMR**	**MVI, DSA-, C4d-**	**Probable AMR**	**TCMR**	**Bordeline**	**No rejection**
**Scenario 4** (Baseline)	**Mixed AMR/TCMR**	0	0	0	0	0	0	0
	**AMR**	0	7	0	0	0	0	0
	**MVI, DSA-, C4d-**	0	0	1	0	0	0	0
	**Probable AMR**	0	2	0	0	0	0	0
	**TCMR**	0	0	0	0	0	0	0
	**Borderline**	0	0	0	0	0	0	0
	**No rejection**	0	0	0	0	0	0	0

Supplementary Table S4. Histological disease course in molecular rejection scenarios. Scenarios 1-3 refer to the descriptions in **Table 3**. Scenario 4: Anti-IL6 targeted treatment in molecular AMR.

		(Follow-up)						
	Total n=15	**Mixed AMR/TCMR**	**AMR**	**MVI, DSA-, C4d-**	**Probable AMR**	**TCMR**	**Bordeline**	**No rejection**
**Molecular negative** (Baseline)	**Mixed AMR/TCMR**	0	0	0	0	1	0	0
	**AMR**	0	2*	0	0	0	0	0
	**MVI, DSA-, C4d-**	0	0	1	0	0	0	0
	**Probable AMR**	0	1	0	2	0	0	0
	**TCMR**	0	0	0	0	0	0	2
	**Borderline**	0	1	0	0	0	0	0
	**No rejection**	0	0	0	1	0	0	4

Supplementary Table S5. Histological disease course in molecular negative cases receiving anti-rejection treatment. Treatment in “no rejection” cases due to tubulitis or other lesions currently classified as no suspicious lesions. *One case receiving clazakizumab.

Supplementary Figure S1. Deduction of the study cohort. All kidney allograft biopsies between July 2018 and January 2025 with histology and MMDx were assessed.

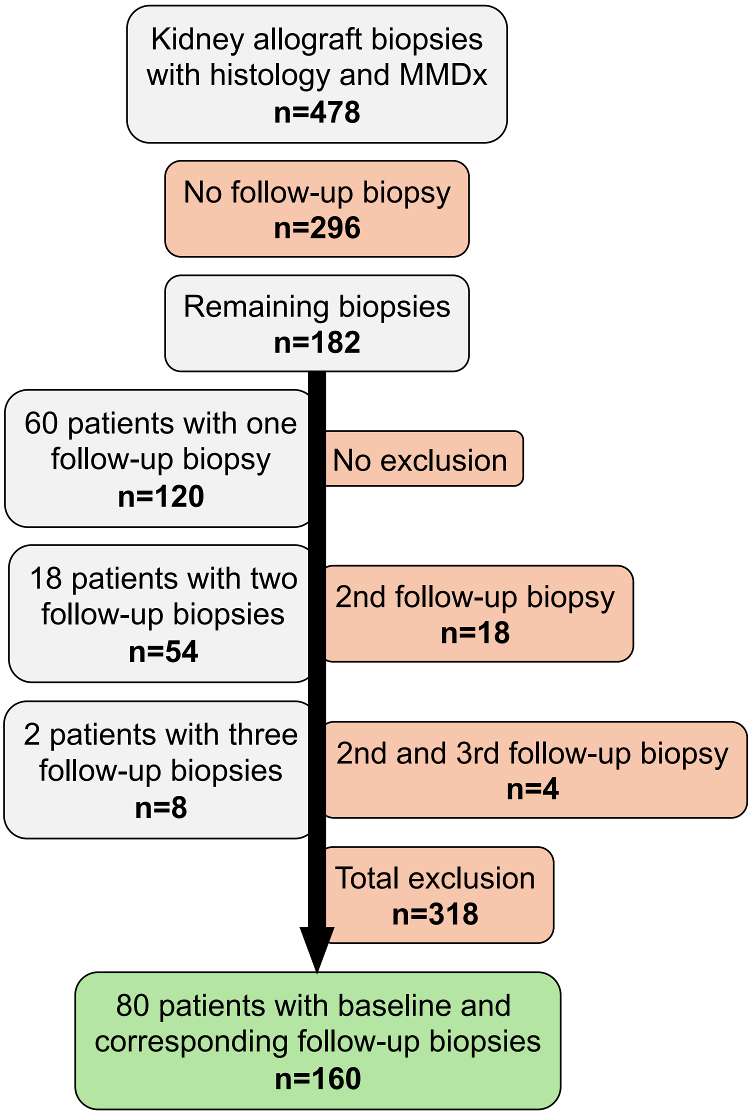

Supplementary Figure S2. Anti-IL6 anti-rejection treatment among molecular AMR. Baseline (BL) and follow-up (FU) biopsies are compared for each scenario using Wilcoxon signed rank-test. The red dashed lines indicate the thresholds level for AMR_{prob}. Median and IQR are demonstrated in blue and the course of each biopsy series below as connected dots.

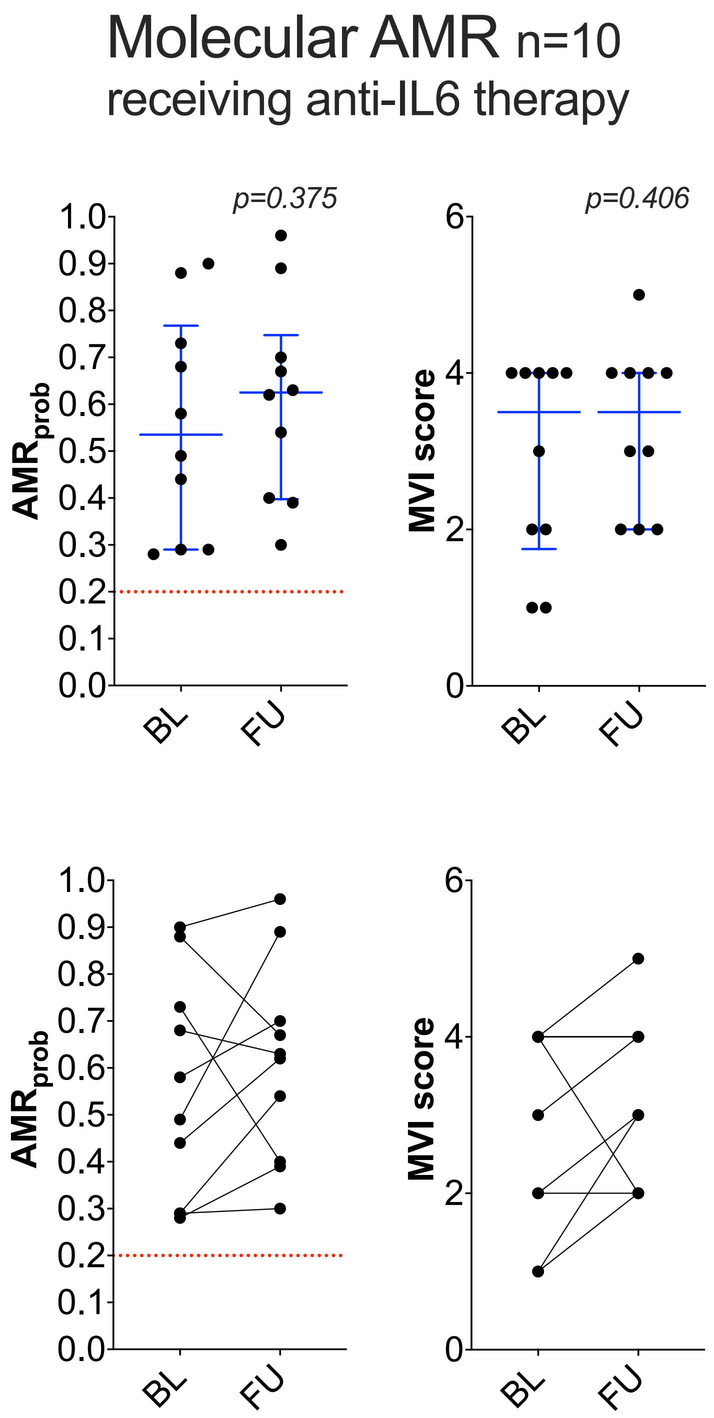

Supplementary Figure S3. Distribution of histological baseline diagnoses along AMR and TCMR classifier activity. The red dashed lines indicate the threshold levels for each score. Baseline biopsies are categorized by color (histological diagnosis).

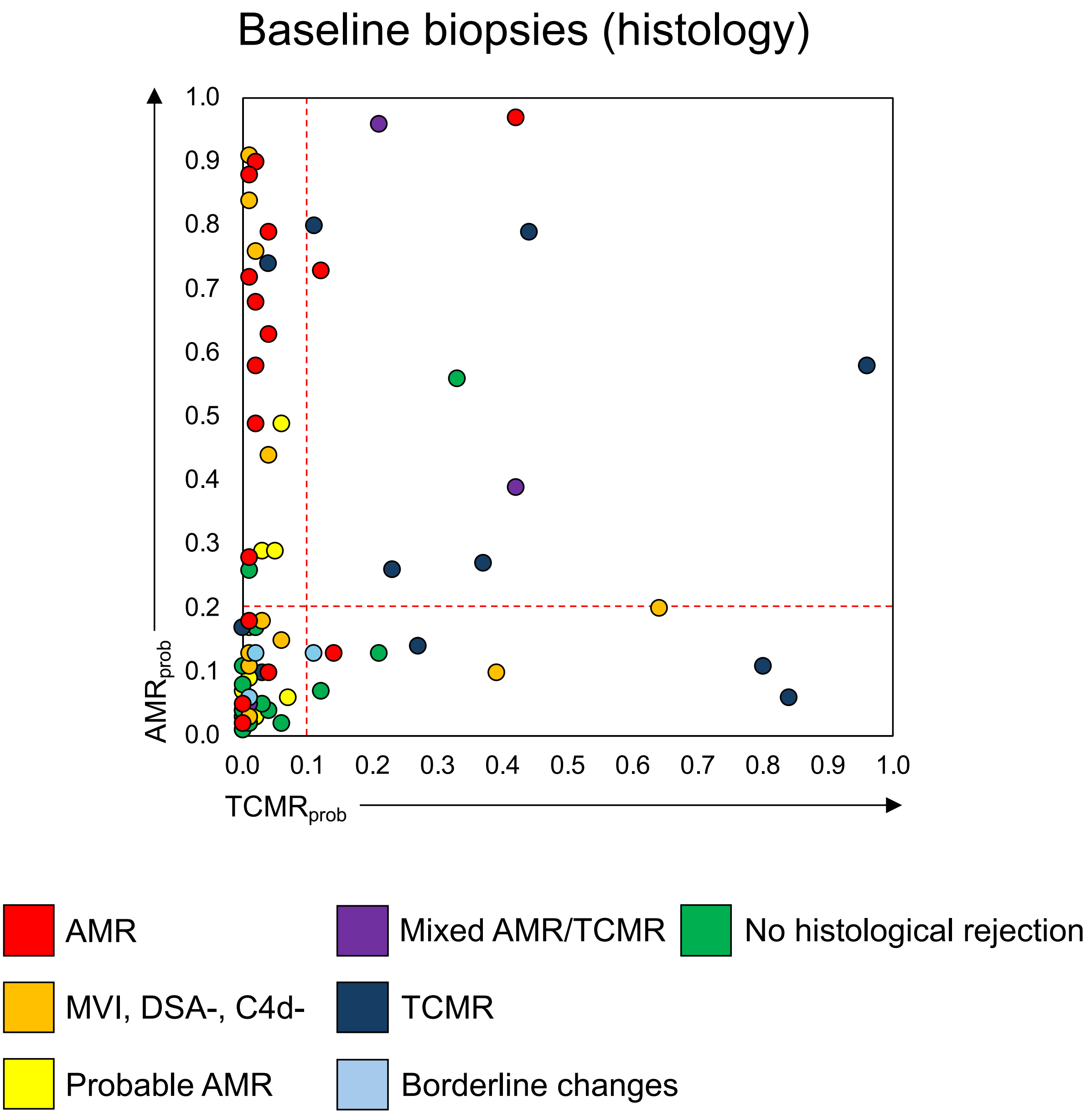

Supplementary Figure S4. Gradual disease course in molecular negative cases receiving anti-rejection treatment. The red dashed lines indicate the threshold levels for each score. Follow-up biopsies reaching molecular rejection states are categorized by color (histological diagnosis of the corresponding baseline biopsy).

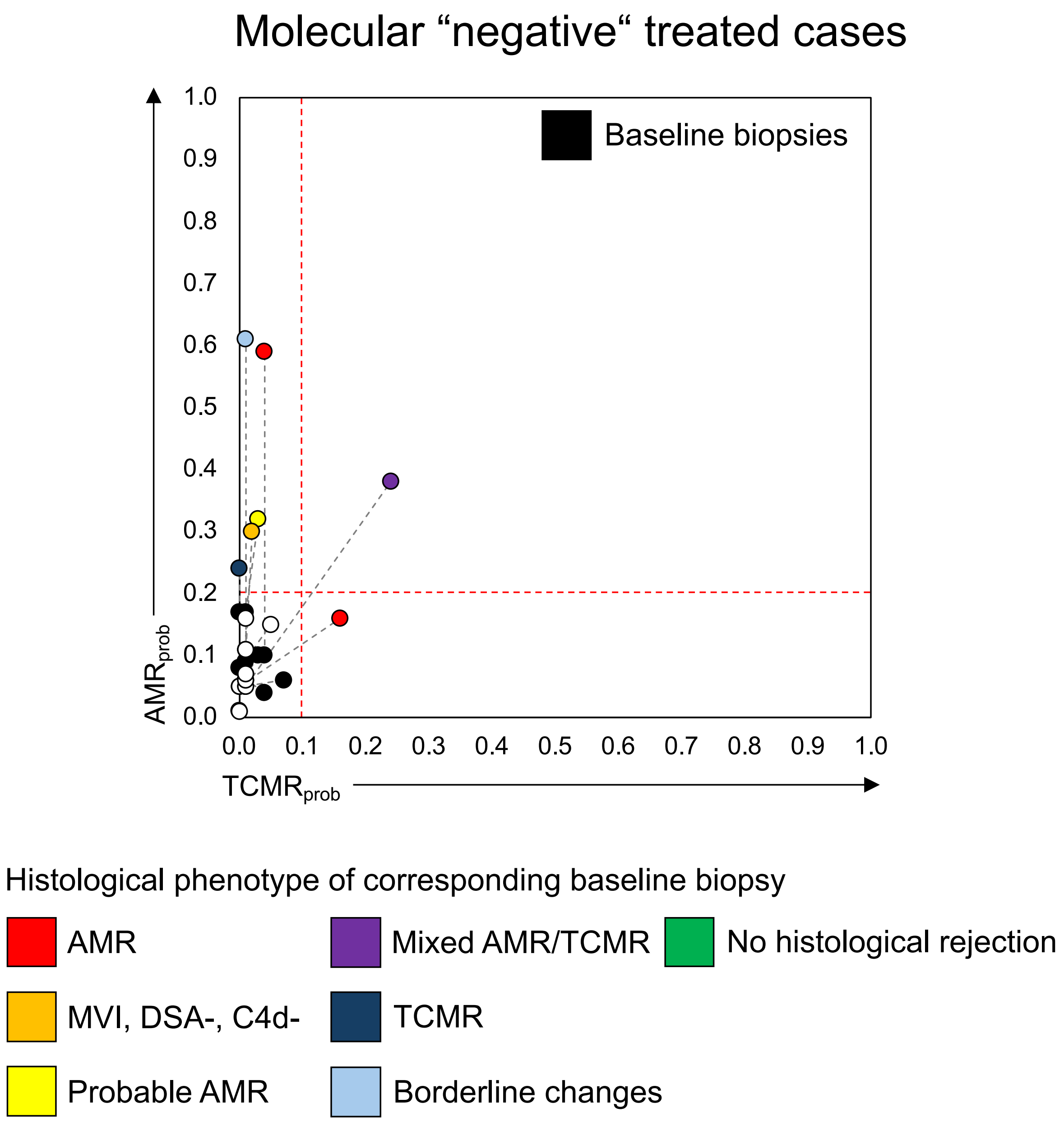

STROBE Statement—Checklist of items that should be included in reports of ***cohort studies***

	Item No	Recommendation
**Title and abstract**	1	(*a*) Indicate the study's design with a commonly used term in the title or the abstract (investigation design with baseline and follow-up biopsies indicated in abstract)
		(*b*) Provide in the abstract an informative and balanced summary of what was done and what was found (main findings are reflected)
**Introduction**		
Background/rationale	2	Explain the scientific background and rationale for the investigation being reported (clinical application of MMDx in a real-life, independent and longitudinal cohort)
Objectives	3	State specific objectives, including any prespecified hypotheses (mentioned in the backgrounds already; previous investigations are mentioned as well)
**Methods**		
Study design	4	Present key elements of study design early in the paper (mentioned in Methods in detail)
Setting	5	Describe the setting, locations, and relevant dates, including periods of recruitment, exposure, follow-up, and data collection (mentioned in detail in the methods: single-center, prospective assessment of biopsies, biopsy process and protocols)
Participants	6	(*a*) Give the eligibility criteria, and the sources and methods of selection of participants. Describe methods of follow-up (inclusion process depicted in Supp. Fig. S1)
		(*b*) For matched studies, give matching criteria and number of exposed and unexposed (no matched study)
Variables	7	Clearly define all outcomes, exposures, predictors, potential confounders, and effect modifiers. Give diagnostic criteria, if applicable (histological and MMDx criteria, assessed molecular signals etc. are mentioned in detail; also limitations section)
Data sources/ measurement	8*	For each variable of interest, give sources of data and details of methods of assessment (measurement). Describe comparability of assessment methods if there is more than one group (biopsy process, DSA, MMDx, and statistical analysis is explained in detail in the methods)
Bias	9	Describe any efforts to address potential sources of bias (indicated in the limitations sector of the discussion)
Study size	10	Explain how the study size was arrived at (demonstrated in Supp. Fig. S1)
Quantitative variables	11	Explain how quantitative variables were handled in the analyses. If applicable, describe which groupings were chosen and why (mentioned in statistical analysis)
Statistical methods	12	(*a*) Describe all statistical methods, including those used to control for confounding (done)
		(*b*) Describe any methods used to examine subgroups and interactions (done)
		(*c*) Explain how missing data were addressed (no missing data for the applied analysis)
		(*d*) If applicable, explain how loss to follow-up was addressed (no loss of follow-up)
		(*e*) Describe any sensitivity analyses (not applied in this study)
**Results**		
Participants	13*	(a) Report numbers of individuals at each stage of study—eg numbers potentially eligible, examined for eligibility, confirmed eligible, included in the study, completing follow-up, and analysed (done)
		(b) Give reasons for non-participation at each stage (not applicable in this study)
		(c) Consider use of a flow diagram (done, Supp. Fig. S1)

Descriptive data	14*	(a) Give characteristics of study participants (eg demographic, clinical, social) and information on exposures and potential confounders (done, baseline characteristics and Supp. Tab. S1)
		(b) Indicate number of participants with missing data for each variable of interest (done, below tables, if applicable)
		(c) Summarise follow-up time (eg, average and total amount) (done, already in abstract)
Outcome data	15*	Report numbers of outcome events or summary measures over time (not applicable)
Main results	16	(*a*) Give unadjusted estimates and, if applicable, confounder-adjusted estimates and their precision (eg, 95% confidence interval). Make clear which confounders were adjusted for and why they were included (not applicable)
		(*b*) Report category boundaries when continuous variables were categorized (done)
		(*c*) If relevant, consider translating estimates of relative risk into absolute risk for a meaningful time period (not applicable)
Other analyses	17	Report other analyses done—eg analyses of subgroups and interactions, and sensitivity analyses (done)
**Discussion**		
Key results	18	Summarise key results with reference to study objectives (done)
Limitations	19	Discuss limitations of the study, taking into account sources of potential bias or imprecision. Discuss both direction and magnitude of any potential bias (done)
Interpretation	20	Give a cautious overall interpretation of results considering objectives, limitations, multiplicity of analyses, results from similar studies, and other relevant evidence (done)
Generalisability	21	Discuss the generalisability (external validity) of the study results (done)
**Other information**		
Funding	22	Give the source of funding and the role of the funders for the present study and, if applicable, for the original study on which the present article is based (done)

*Give information separately for exposed and unexposed groups.

Note: An Explanation and Elaboration article discusses each checklist item and gives methodological background and published examples of transparent reporting. The STROBE checklist is best used in conjunction with this article (freely available on the Web sites of PLoS Medicine at http://www.plosmedicine.org/, Annals of Internal Medicine at http://www.annals.org/, and Epidemiology at http://www.epidem.com/). Information on the STROBE Initiative is available at http://www.strobe-statement.org.
